# Identification and characterization of MYH9 locus for high efficient gene knock-in and stable expression in mouse embryonic stem cells

**DOI:** 10.1371/journal.pone.0192641

**Published:** 2018-02-13

**Authors:** Tanbin Liu, Yi Hu, Shiyin Guo, Lei Tan, Yang Zhan, Lingchen Yang, Wei Liu, Naidong Wang, Yalan Li, Yingfan Zhang, Chengyu Liu, Yi Yang, Robert S. Adelstein, Aibing Wang

**Affiliations:** 1 Lab of Animal Models and Functional Genomics (LAMFG), The Key Laboratory of Animal Vaccine & Protein Engineering, College of Veterinary Medicine, Hunan Agricultural University (HUNAU), Changsha, Hunan, China; 2 College of Food Science and Technology, HUNAU, Changsha, Hunan, China; 3 Lab of Functional Proteomics (LFP), The Key Laboratory of Animal Vaccine & Protein Engineering, College of Veterinary Medicine, HUNAU, Changsha, Hunan, China; 4 Lab of Molecular Cardiology (LMC), National Heart, Lung, and Blood Institute (NHLBI)/National Institutes of Health (NIH), Bethesda, MD, United States of America; 5 Transgenic Core, NHLBI/ NIH, Bethesda, MD, United States of America; University of Texas at Austin Dell Medical School, UNITED STATES

## Abstract

Targeted integration of exogenous genes into so-called safe harbors/friend sites, offers the advantages of expressing normal levels of target genes and preventing potentially adverse effects on endogenous genes. However, the ideal genomic loci for this purpose remain limited. Additionally, due to the inherent and unresolved issues with the current genome editing tools, traditional embryonic stem (ES) cell-based targeted transgenesis technology is still preferred in practical applications. Here, we report that a high and repeatable homologous recombination (HR) frequency (>95%) is achieved when an approximate 6kb DNA sequence flanking the MYH9 gene exon 2 site is used to create the homology arms for the knockout/knock-in of diverse nonmuscle myosin II (NM II) isoforms in mouse ES cells. The easily obtained ES clones greatly facilitated the generation of multiple NM II genetic replacement mouse models, as characterized previously. Further investigation demonstrated that though the targeted integration site for exogenous genes is shifted to MYH9 intron 2 (about 500bp downstream exon 2), the high HR efficiency and the endogenous MYH9 gene integrity are not only preserved, but the expected expression of the inserted gene(s) is observed in a pre-designed set of experiments conducted in mouse ES cells. Importantly, we confirmed that the expression and normal function of the endogenous MYH9 gene is not affected by the insertion of the exogenous gene in these cases. Therefore, these findings suggest that like the commonly used ROSA26 site, the MYH9 gene locus may be considered a new safe harbor for high-efficiency targeted transgenesis and for biomedical applications.

## Introduction

Transgenic mouse models are one of the most powerful tools for determining the functions of interesting genes. Additionally, integrative gene transfer is also widely used for bioproduction, drug screening, and therapeutic applications. The insertion of foreign DNA into a chromosome can be achieved either in a site-specific or random genomic integration manner. Notably, it has been widely believed that targeted integration at predetermined sites is preferred over random insertion in order to prevent interference with transgene expression, insertional mutagenesis, activation of neighboring genes, as well as cell toxicity [1–3]. In particular, the gene knock-in approach is frequently utilized to produce human disease models, including humanized animals [4–7].

A gene targeted integration or knock-in strategy refers to the insertion of DNA into a precise chromosomal site through homologous recombination (HR). Mouse embryonic stem (ES) cells, along with stringent selection methods following gene targeting by HR, are commonly used to generate transgenic mice [8]. Conventional gene targeting methods in mammalian cells including ES cells, are time-consuming and laborious mainly due to the low HR frequency [9]. Though the appearance of novel genome editing technologies including Zinc Finger Nucleases (ZFN), Transcription Activator-Like Effector Nucleases (TALEN), Clustered Regularly Interspaced Short Palindromic Repeats (CRISPR)/CRISPR-associated protein9 or CRISPR/Cas9) facilitates the improvement of low gene targeting efficiency [10–13], these tools are also accompanied by some issues, such as off-target effects [14], the time-consuming and laborious design necessary for generation of ZFN and TALEN, and the low efficiency of CRISPR/Cas9 in the knock-in of long DNA fragment [15]. Therefore, conventional ES cell-based transgenesis technology still has its applied uses for the resolution of these shortcomings with these novel gene editing tools, especially for studies on the ES cell differentiation program and for creating mouse strains expressing Cre recombinase, etc [16–19]. Targeted integration or “knock-in” also involves the selection of a precise genome locus or so-called safe harbor into which the exogenous gene is inserted, thereby circumventing potential “positional effects” and avoiding the interference in the genome [20–22]. A well-known example of such sites is the Rosa26 locus which is widely used for targeted transgenesis in mice, mainly due to the stable, ubiquitous and strong expression of the exogenous gene, and the high frequency of gene targeting in murine ES cells with no observed side-effect on the genome at this position [23–24]. However, the number of ideal genome sites for gene knock-in is still limited, and more permissive loci other than Rosa26 need to be identified in order to provide practical options for genetic engineering.

In an effort to explore nonmuscle myosin II (NM II) isoform- and domain-specific functions as well as the mechanisms underlying MYH9 related disease (MYH9-RD), we produced several targeted transgenic mouse lines based on gene targeting in mouse embryonic stem (ES) cells using the same genetic replacement strategy. Briefly, the endogenous MYH9 gene encoding nonmuscle myosin heavy chain IIA (NHMC IIA) was disrupted by the targeted insertion of cDNAs expressing NMHC IIB, chimeric or mutant NMHC IIs, in which each expression cassette was placed under the control of the endogenous MYH9 promoter. Consequently, mutant mice lacked endogenous NM IIA but expressed the knock-in protein [25–26]. In the course of screening the desired ES clones, we surprisingly found that the gene targeting efficiency at the MYH9 gene exon 2 site (95%) was much higher than that at the Rosa26 locus (25%) based on the similarity of the approach used [27]. This finding prompted us to examine the potential of the MYH9 gene locus (e.g. exon 2 and intron 2 region) as a novel safe harbor for targeted transgenesis. In this study we report, for the first time, the high gene targeting frequency at the MYH9 gene locus in mouse ES cells. This feature could be used to generate other genetic replacement mouse models for studying the functions of different NM II isoforms and further understanding the underlying mechanisms of MYH9-RD. Importantly, we provide evidence to show that the exogenous gene is robustly expressed mainly depending on the MYH9 promoter or its fused promoter when it is integrated into the MYH9 locus, while the high gene targeting efficiency and the normal expression of the endogenous MYH9 gene are maintained. These data suggest that the MYH9 locus is valuable as an alternative safe harbor for targeted transgenesis.

## Materials and methods

This study was approved by the Animal Ethics Committee of Hunan Agricultural University, Hunan, China. No animal experiments were involved, no ethic permits were therefore required for the described work, which complied with all relevant regulations. All mouse procedures carried out at the NHLBI were carried out in accordance with NHLBI ACUC guidelines.

### Generation of targeting constructs

**Fig 1:**
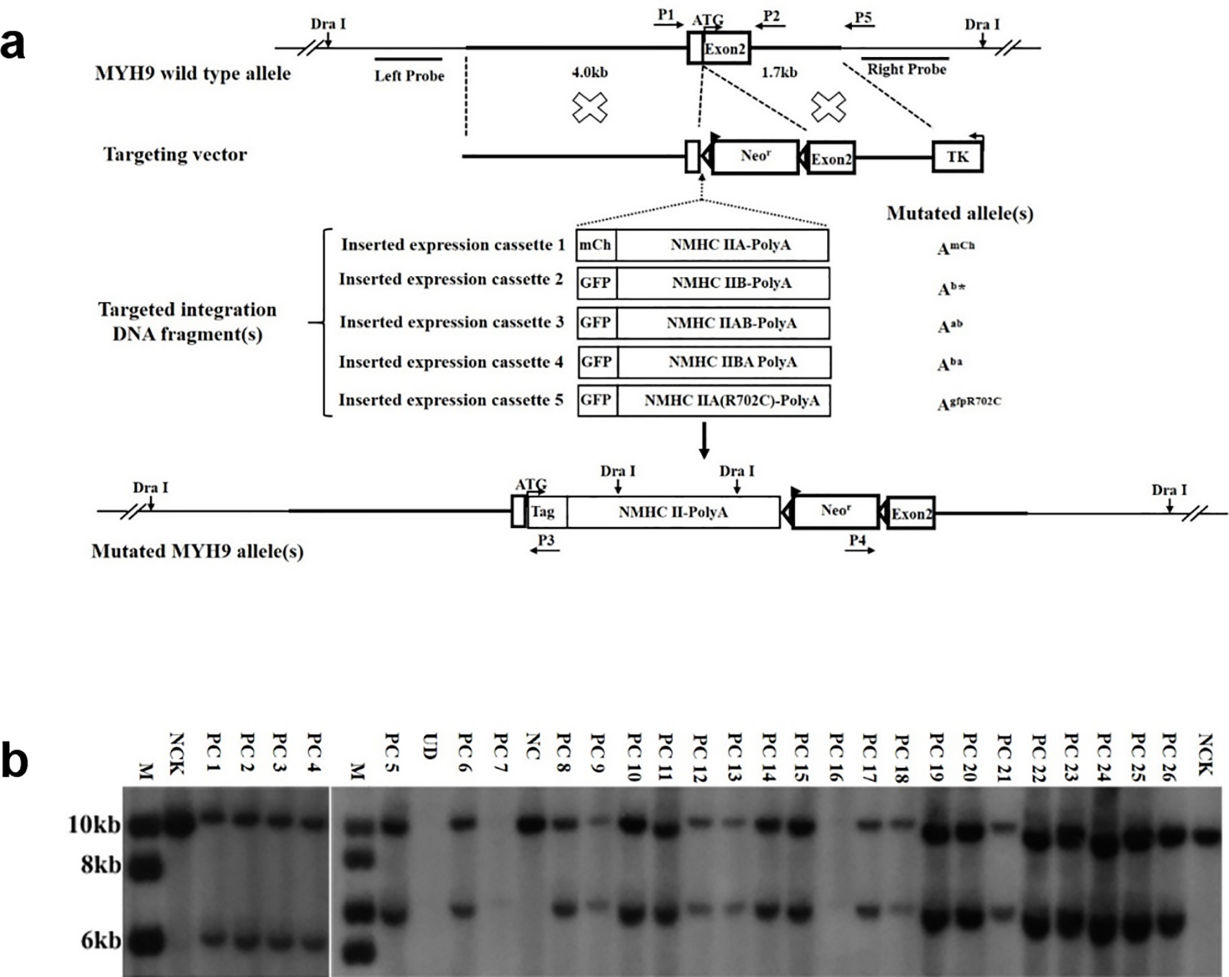
Targeted exogenous gene(s) integration into the mouse MYH9 gene exon 2 site. (a). Schematic demonstrating the strategy for targeted integration of exogenous gene(s), wild type (WT) MYH9 gene allele; gene targeting vector, targeted integration DNA fragment(s) (cassette 1–5), as well as resultant mutated allele(s). Probes for Southern blot and primers for PCR are also shown and described previously. (b). Southern blot screening of the genomic DNAs from the mouse A^+^/A^mCh^ ES cell clones using the Right Probe. The mutated allele shows a 6.0kb band, while WT shows a 9.7kb band. M, Marker; NCK: Negative Control; PC, Positive Clone; UD, Undetermined; NC, Negative Clone.

**Fig 2:**
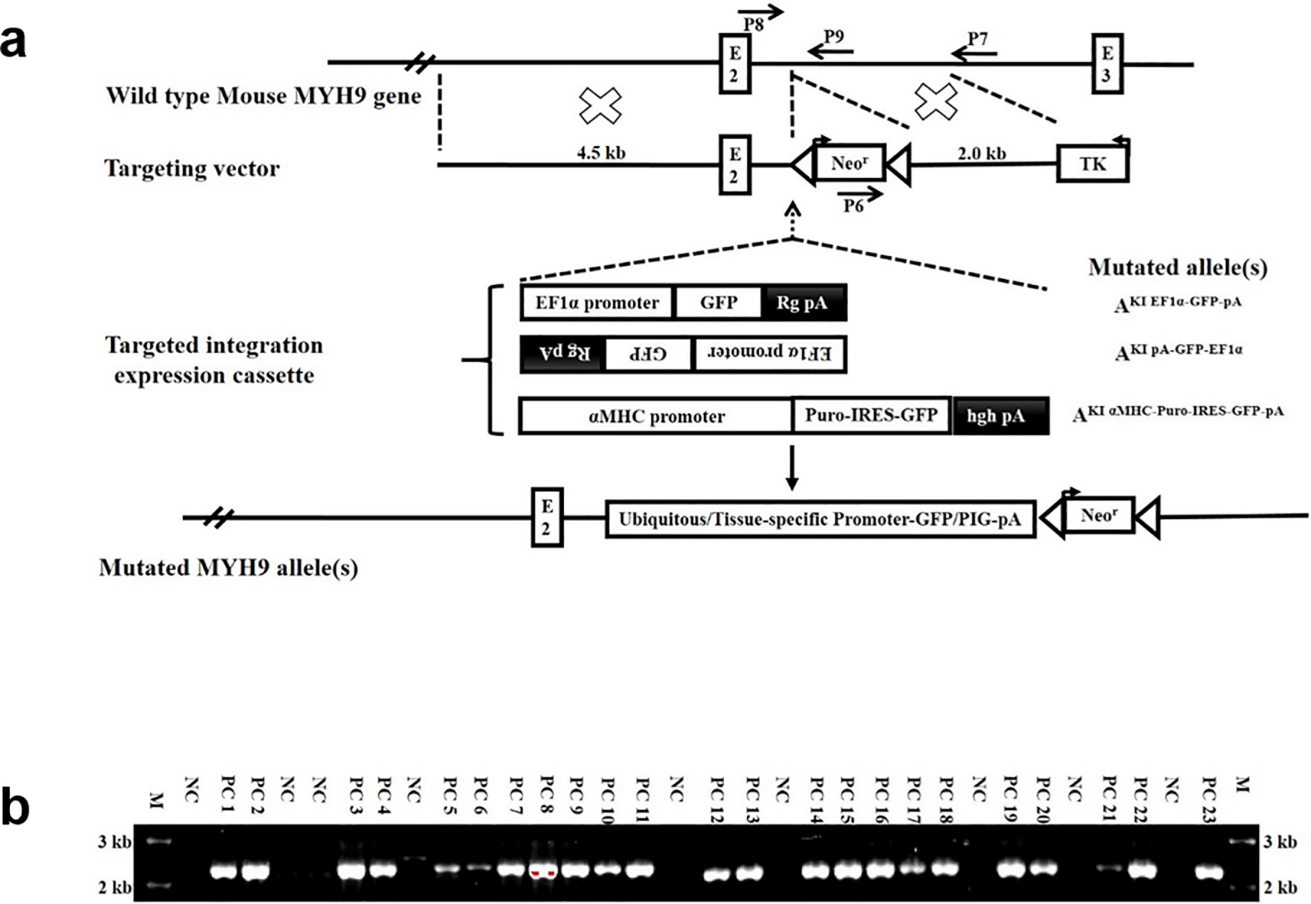
Targeted exogenous gene(s) integration at mouse MYH9 intron 2 site. (a). Schematic demonstrating the strategy for targeted integration of exogenous gene(s), wild type (WT) MYH9 gene allele (top); gene targeting vector, targeted integration expression cassette(s), as well as resultant mutated allele(s) (bottom). Primers for PCR screening are also shown (P6-P9). (b). PCR screening of the genomic DNAs from mouse knock-in Neomyocin (Neo^r^) ES cell clones using the primer pair P6+P7. The mutated allele yielded a 2.2kb band, while the WT allele yielded no band. M, Marker; PC, Positive Clone; NC, Negative Clone.

All knock-in constructs targeting exon 2 of the MYH9 gene have been described previously [25–26] and shown Fig 1A as well. The knock-in vectors targeting intron 2 of MYH9 are shown in Fig 2A. In detail, the 5’ 4.5kb and the 3’ 2.0kb arms were amplified from a 129/Sv genomic BAC clone and cloned into the vector mpNTKV-LoxP, as described previously [25], and the resulting vector was named Knock-in Neo. The EF1α-GFP-rGlobin polyA expression cassette was amplified by PCR using pEF-GFP (Addgene) as the template and cloned into the Knock-in Neo vector, and the resultant vectors were called Knock-in EF1α-GFP-pA and Knock-in pA-GFP-EF1α according to the inserted orientation, respectively. To create a Knock-in αMHC-Puro-IRES-GFP-pA vector, the Puro-IRES-GFP fragment was amplified by PCR using pMSCV Puro-IRES-GFP (Addgene) as the template, and cloned into the pJG/αMHC vector (Addgene), then the αMHC-Puro-IRES-GFP-hgh polyA fragment was excised for the insertion into the Knock-in Neo vector. Primers used in amplifying DNA fragments are listed in the Supporting Information S1 Table. Nucleotide sequences of the cloned DNA fragments were confirmed in all cases by sequencing. All knock-in vectors were linearized by restriction enzymes before electroporation.

### Mouse embryonic stem cells (mESCs) culture and electroporation

The V6.5 ES cell line derived from C57BL/6 and 129S6SvEv F1 hybrid mice was cultured and electroporated with linearized targeting vectors as before [25–26], followed by drug selection using 200–800μg/ml G418 and 200μM ganciclovir. Drug resistant colonies were picked and expanded for genomic DNA preparation.

### Identification of recombination events by Southern blotting and PCR screening

HR events occurring at MYH9 exon 2 site were analyzed by Southern Blot; the probes and primers used were reported previously [25–26] and are indicated in Fig 1A. Additionally, HR events occurring at the MYH9 intron 2 site were identified by long-range PCR using genomic DNA prepared with a genomic DNA purification kit (Promega) as template and Platinum Taq High Fidelity DNA Polymerase (Invitrogen) for amplification. The forward primer in the 3’ end of neomycin and the reverse primer located outside the 3’ short arm, are listed in S1 Table. The PCR reaction conditions were: initial denaturation at 94°C for 3 min, 30 cycles of denaturation at 94°C for 0.5min, annealing at 60°C for 0.5min and extension at 68°C for 2.2 min, and a final step of 68°C for 10 min. The PCR products were analyzed by electrophoresis in 1.0% agarose, the targeted allele yielded a 2.2kb band while wildtype allele produced no band. Several randomly selected PCR-amplified products were excised, extracted and cloned into T-easy vector for sequencing (Promega).

### Observation and differentiation of mESCs

Wild type and the desired targeted ES clones with forward or reverse orientation of the EF1α-GFP cassette, were routinely maintained and passaged as before [25–26]. ES cells passaged at least three times were observed using fluorescence microscope and randomly selected fields were imaged.

Wild type and ES clones targeted with an αMHC-Puro-IRES-GFP cassette were routinely cultured and differentiated into cardiomyocytes as previously described with minor modification [28]. Briefly, embryoid bodies from A^+^/A^+^ and A^+^/A^KI αMHC-Puro-IRES-GFP-pA^ ES cells were formed using the hanging drop method, followed by successive activin A and BMP4 treatment, and then treated with puromycin for the indicated time period and concentration. GFP signals from embryoid bodies were recorded to show the extent of differentiation. Beating of differentiated ES cells was observed on day 8 and thereafter.

### Immunoblot analysis

Immunoblot analysis was performed as described previously [25–26]. Briefly, total proteins were extracted by directly lysing cultured ES cells in 1×RIPA buffer (Santa Cruz) supplemented with protease inhibitor cocktail (Roche Diagnostics), separated by SDS-PAGE and transferred onto a PVDF membrane (Invitrogen), immunoblotted with anti-NMHC IIA or anti-β-actin and anti-GFP antibodies, followed by incubation with peroxidase-conjugated goat anti-rabbit/mouse secondary IgGs (Abcam), and developed using horseradish peroxidase substrate (Thermo Fisher) to visualize the protein bands.

## Results

### High-efficiency gene targeting at the MYH9 gene exon 2 site in mouse ES cells

To explore isoform- and domain-specific functions of nonmuscle myosin II (NM II) in development and to generate mouse models with disease-causing point mutations in NM IIA to mimic MYH9-related diseases in human, we adopted an ES cell-based method and genetic replacement strategy to generate multiple mouse models. As indicated in Fig1A and described in our previous studies [25–26], the genetic replacement strategy simultaneously involved the disruption of the first coding exon of the endogenous MYH9 gene and the targeted integration of exogenous expression cassette(s) encoding GFP-tagged human nonmuscle myosin heavy chain IIB (NMHC IIB), GFP-fused chimeric NMHC IIAB or IIBA (the N-terminal domain of NMHC IIA followed by the C-terminal domain of IIB, or vice versa), or GFP-tagged NMHC IIA with an R702C point mutation. Similarly, mCherry-tagged NMHC IIA was inserted into the same site of the MYH9 locus to function as a control, and to monitor effectiveness of the strategy. Thus, totally 5 individual gene targeting events were reported at the MYH9 exon 2 site in this study, and the targeted alleles were called A^b^*, A^ab^, A^ba^, A^gfpR702C^, A^mCh^, respectively, as indicated in Fig 1A.

Following separate electroporation of the different targeting constructs into mouse ES cells and drug screening, random ES colonies were picked and expanded for genomic DNA extraction. Southern blotting was used to identify potential positive ES clones. As summarized in Table 1, the results indicated several unique features: i). except for the construct used to generate the A^ab^ allele, a >90% frequency of homologous recombination (HR) was observed for the other 4 constructs. Though 192 ES colonies were picked for each construct, about one seventh of them in each case were screened by Southern blot for positive ES cell clones because of this high HR efficiency. For instance, 26 randomly selected clones out of 27 clones were identified to be the desired A^+^/A^mCh^ cells (Fig 1B); ii) only one mutated allele was found in all cases; iii) no off-target integration of the targeting vector(s) was detected; iv) importantly, this high HR efficiency was repeatable and reliable in three independent experiments. To our knowledge, this >90% HR frequency is the highest among similar gene targeting experiments in mouse ES cells so far.

**Table 1:** Homologous recombination (HR) efficiency of gene targeting at MYH9 exon 2 site.

Genotype	Positive/Screened clones	HR efficiency (%)
A^+^/A^mCh^	26/27	96.3
A^+^/A^b^*	27/28	96.4
A^+^/A^ab^	18/26	69.2
A^+^/A^ba^	24/25	96.0
A^+^/A^gfpR702C^	17/19	89.5

Note

mCh: mCherry-tagged NMHC IIA

b*: GFP-tagged NMHC IIB

ab: GFP-tagged chimeric NMHC IIAB

ba: GFP-tagged chimeric NMHC IIBA

gfpR702C: GFP-tagged NMHC IIA with R702C mutation. The representations of these symbols can also be found in the references [25, 26].

### Genetic replacement mouse models advance our understanding of NM II functions in development and disease

As we expected, the targeted insertion of predesigned expression cassettes into the MYH9 `exon 2 site led to the ablation of one allele of the endogenous MYH9 gene and the expression of fluorescence tagged NMHC IIs both in ES cells and the mice derived from these ES cell lines [25–26]. Several characteristics of the genetic replacement strategy used in our studies should be noted: i) either A^+^/A^mCh^ or A^mCh^/A^mCh^ cells or mice were indistinguishable from their wild type littermates, demonstrating that the method adopted is feasible and reasonable, and the phenotypes observed in the mutant mice are not a consequence of genetic manipulations of the MYH9 locus; ii) whether the neomycin selection marker, integrated with NMHC IIs expression cassettes was maintained or excised, the expression of these exogenous NMHC IIs was not significantly affected. Importantly, the expression levels of these exogenous NMHC IIs were close to the physiological level of the endogenous MYH9 gene, suggesting that the expression of these promoter-less expression cassettes are indeed under the control of the MYH9 gene context, though the endogenous MYH9 gene promoter is located 28kb upstream from the first coding exon 2; iii) interestingly, either the absence of one allele (e.g. A^+^/A^-^) or mutation of one allele (e.g. A^+^/A^b^*, A^+^/A^ab^, A^+^/A^ba^, A^+^/A^mCh^) of the MYH9 gene in cells or in mice did not result in detectable abnormalities [25,29], however, A^+^/A^gfpR702C^ mice having a point mutation R702C on NMHC IIA showed the phenotypes of MYH9-RD observed in humans [26], as also verified by *in situ* R702C NMHC IIA mutated mice [30]. Detailed information about these mice and our understanding of the functions of NM IIs in development and disease has been published. [25–26,31].

### High frequency gene targeting at the MYH9 intron 2 site in mouse ES cells

Next, we explored whether the MYH9 gene locus can be used as a safe harbor for targeted exogenous gene integration. To achieve this, several questions were addressed. First, though single allele absence or replacement in MYH9 did not cause any obvious consequences, could any disturbance of the endogenous gene be avoided? Second, to maintain endogenous MYH9 gene expression, could exogenous expression cassette(s) be inserted into the intron (e.g. intron 2) while preserving the high HR frequency. Third, as indicated above, although the expression of the promoterless expression cassette(s) is regulated by the endogenous MYH9 gene context, could a targeted exogenous gene(s) be controlled by its fused ubiquitous or tissue-specific promoter. To investigate these questions, we created four sets of targeting constructs as indicated in Fig 2A.

We initially introduced a construct containing a 4.5 kb 5’ homology arm, the selection marker neomycin and a 2.0 kb 3’ homology arm into mouse ES cells, thus, only the neomycin cassette would be inserted into the intron 2 site of the MYH9 gene following HR. Twenty-three out of 32 randomly selected ES clones were confirmed to be targeted correctly by PCR using a primer located at the 3’ end of the neomycin cassette (P6) and another primer outside of the 3’ arm (P7, Fig 2A). As shown in Fig 2B, ES clones with targeted integration of neomycin displayed a 2.2 kb PCR band, whereas negative clones had no band. To further verify this targeted integration event, the 2.2kb PCR band from 3 samples was excised and cloned into T-easy vector and plasmids from 10 randomly selected bacterial clones were prepared and sequenced. The results of sequencing reflected site-specific integration of neomycin at the MYH9 intron 2 site.

Recognizing the high HR efficiency at the MYH9 gene intron 2 site, we therefore tried to incorporate exogenous cassettes into the targeting constructs (Fig 2A). These included a cassette expressing GFP under the control of the EF1α (ubiquitous) promoter and its counterpart which has the opposite transcription direction, a cassette expressing puromycin and GFP under the control of αMHC (cardiac-specific) promoter, as indicated in Fig 2A. When these three new constructs were introduced into mouse ES cells, approximately 70% HR frequency was observed for each construct (Table 2). In sum, 16–19 out of 24 randomly picked ES clones were identified to be the desired ones using the PCR method described above. Likewise, a single copy exogenous cassette(s) integration into an allele of MYH9 gene was confirmed. The 5’ and 3’ ends of the integration event from 3 clones per construct were sequenced and verified. The resulting mutant alleles in MYH9 intron 2 were called A^KI EF1α-GFP-pA^, A^KI pA-GFP-EF1α^, A^KI αMHC-Puro-IRES-GFP-pA^, respectively (Fig 2A).

**Table 2:** HR efficiency of gene targeting at MYH9 intron 2 site.

Targeting vector(s)	Positive/Screened clones	HR efficiency(%)
Knock-in Neo	23/32	71.8
Knock-in EF1α-GFP-pA	17/24	70.8
Knock-in pA-GFP-EF1α	19/24	79.2
Knock-in αMHC-Puro-IRES-GFP-pA	16/24	66.7

### The expression of targeted integration exogenous gene(s) relies on their fused promoter

**Fig 3:**
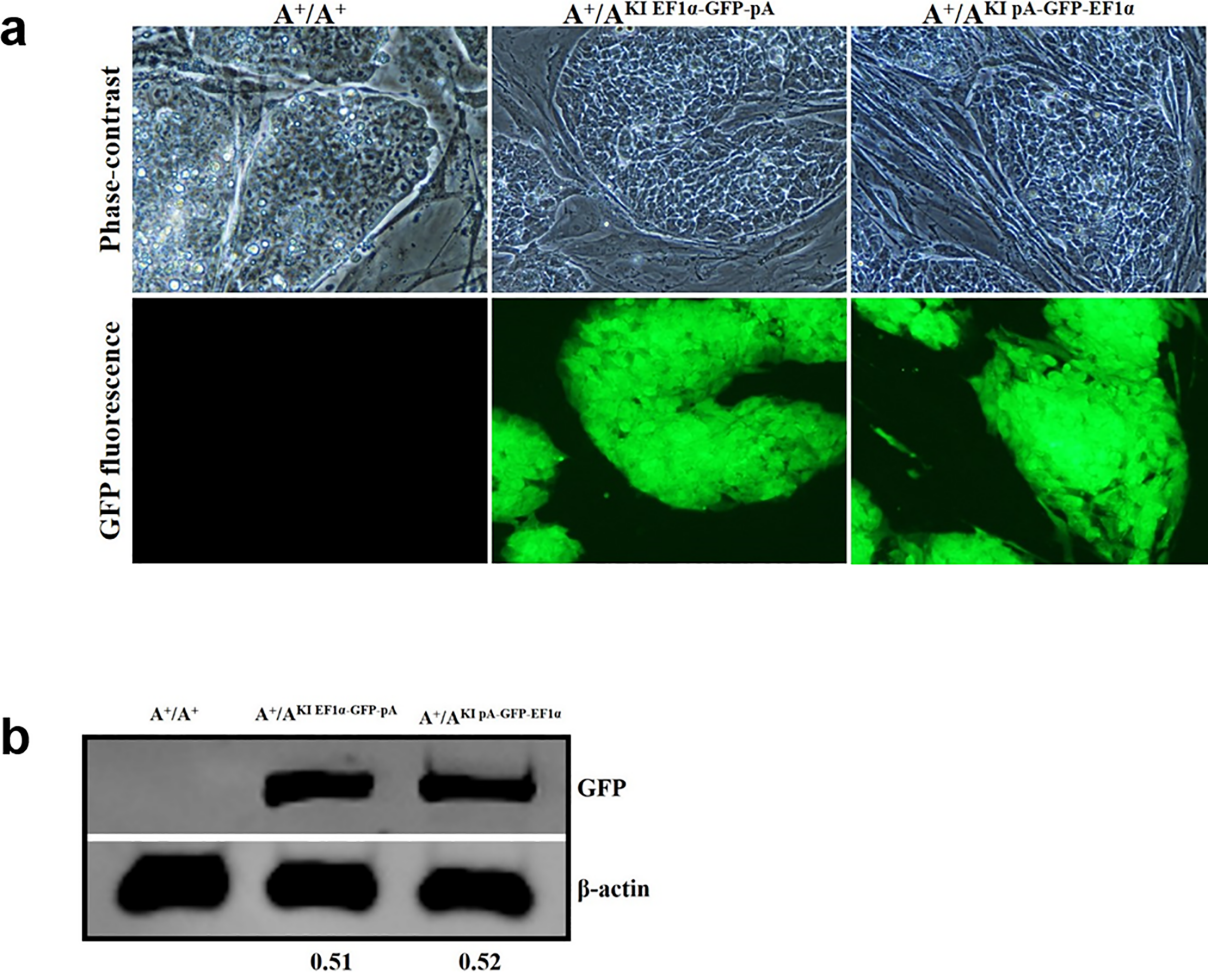
The expression of ubiquitous promoter-controlled exogenous gene(s) integrated at the mouse MYH9 intron 2 site. (a). GFP expression under the control of the EF1α promoter in mouse ES cells was monitored by fluorescent intensity. Shown are phase contrast (upper panels, 20X objective) and GFP fluorescence images (lower panels, 20X objective) of different genotypes of mouse ES cells. Note the similar GFP intensity of A^+^/A^KI EF1α-GFP-pA^ and A^+^/A^KI pA-GFP-EF1α^ cells and the lack of GFP signal in wild type cells at the same microscope setting. (b). Immunoblot analysis of targeted knock-in GFP from different genotypes of mouse ES cells probed with anti-GFP and anti-β-actin. The expression of β-actin was used as the loading control. The ratios of GFP to β-actin are shown below the blot. Representative results are from three repeats.

Having obtained the desired ES clones with the insertion of an exogenous cassette into the MYH9 intron 2, we next examined the expression of these inserted genes. Randomly selected A^+^/A^KI EF1α-GFP-pA^ and A^+^/A^KI pA-GFP-EF1α^, together with A^+^/A^+^ ES clones, were cultured and GFP fluorescence intensity was used to monitor the expression level. As indicated in Fig 3A, a strong GFP signal was seen in the A^+^/A^KI EF1α-GFP-pA^ and A^+^/A^KI pA-GFP-EF1α^ ES cells but not in the A^+^/A^+^ ones. To further quantitate this observation, we performed immunoblot assays to determine the GFP expression levels in these different cells. The result showed an approximately equal amount of GFP expression in A^+^/A^KI EF1α-GFP-pA^ and A^+^/A^KI pA-GFP-EF1α^ ES cells (Fig 3B). These data demonstrate that the exogenous gene targeted integration into the MYH9 intron 2 is robustly expressed, and furthermore, the expression level relies on the driven ubiquitous promoter (EF1α) rather than the transcription direction and the MYH9 gene context.

**Fig 4:**
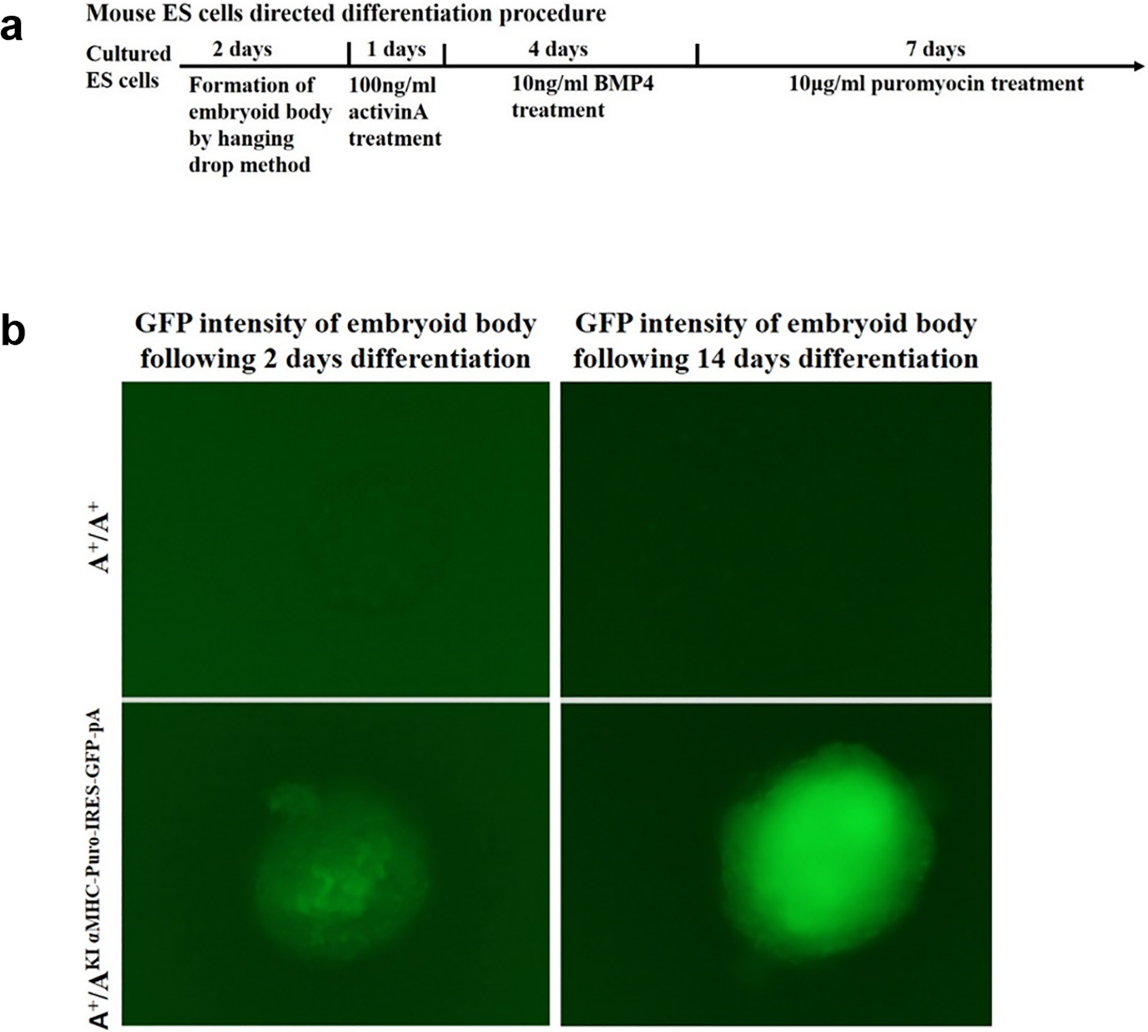
The expression of a cardiac-specific promoter (αMHC)-controlled exogenous puromyocin/GFP targeted integration at the mouse MYH9 gene intron 2 site. (a). Schematic of the timeline of the directed ES cells differentiation into cardiomyocytes. (b). ES cell differentiation into cardiomyocytes was monitored by GFP fluorescence intensity. Shown are GFP fluorescence images (10X objective) of different genotypes of mouse ES cells at different time points. Note the gradually enhanced GFP intensity in A^+^/A^KI αMHC-Puro-IRES-GFP-pA^ cells and lack of GFP signal in wild type cells.

Likewise, the expression of cardiac-specific promoter-controlled puromycin and GFP was analyzed in targeted ES clones. A diagram demonstrating the procedure for ES cells differentiation into cardiomyocytes is shown in Fig 4A, with minor modification from a previous report [28]. Briefly, embryoid bodies from A^+^/A^+^ and A^+^/A^KI αMHC-Puro-IRES-GFP-pA^ ES cells were formed by the hanging drop method, followed by successive activinA and BMP4 treatment, and then treated with puromycin for the indicated time period. The GFP signal from the embryoid bodies was recorded to show the extent of differentiation. Not surprisingly, weak GFP fluorescence was observed in A^+^/A^KI αMHC-Puro-IRES-GFP-pA^ embryoid bodies but not in A^+^/A^+^ ones following 2 days of differentiation. With an extension of differentiation time, the GFP fluorescence in A^+^/A^KI αMHC-Puro-IRES-GFP-pA^ embryoid bodies gradually increased and reached a plateau level following 14 days of differentiation, while A^+^/A^+^ embryoid bodies were completely apoptotic at this time point due to the absence of the Puro-resistant gene (Fig 4B). Of note, after 7 days of puromycin treatment, all A^+^/A^KI αMHC-Puro-IRES-GFP-pA^ embryoid bodies consisted of strongly beating GFP^+^ clusters of cardiac myocytes (S1 Video). Therefore, these results demonstrate that the cardiac-specific αMHC promoter independently exerted its function and robustly drove the expression of a fused gene(s) within the MYH9 intron context, comparable to the random integration of the same expression cassette in a previous study [28]. Collectively, these data suggest that the expression of targeted integration cassette(s) at the MYH9 gene intron 2 site relies on the fused promoter, and has no relation to the inserted direction and the MYH9 gene context.

### The expression of the endogenous MYH9 gene is not affected by the insertion of exogenous gene(s) at the intron 2 site

**Fig 5:**
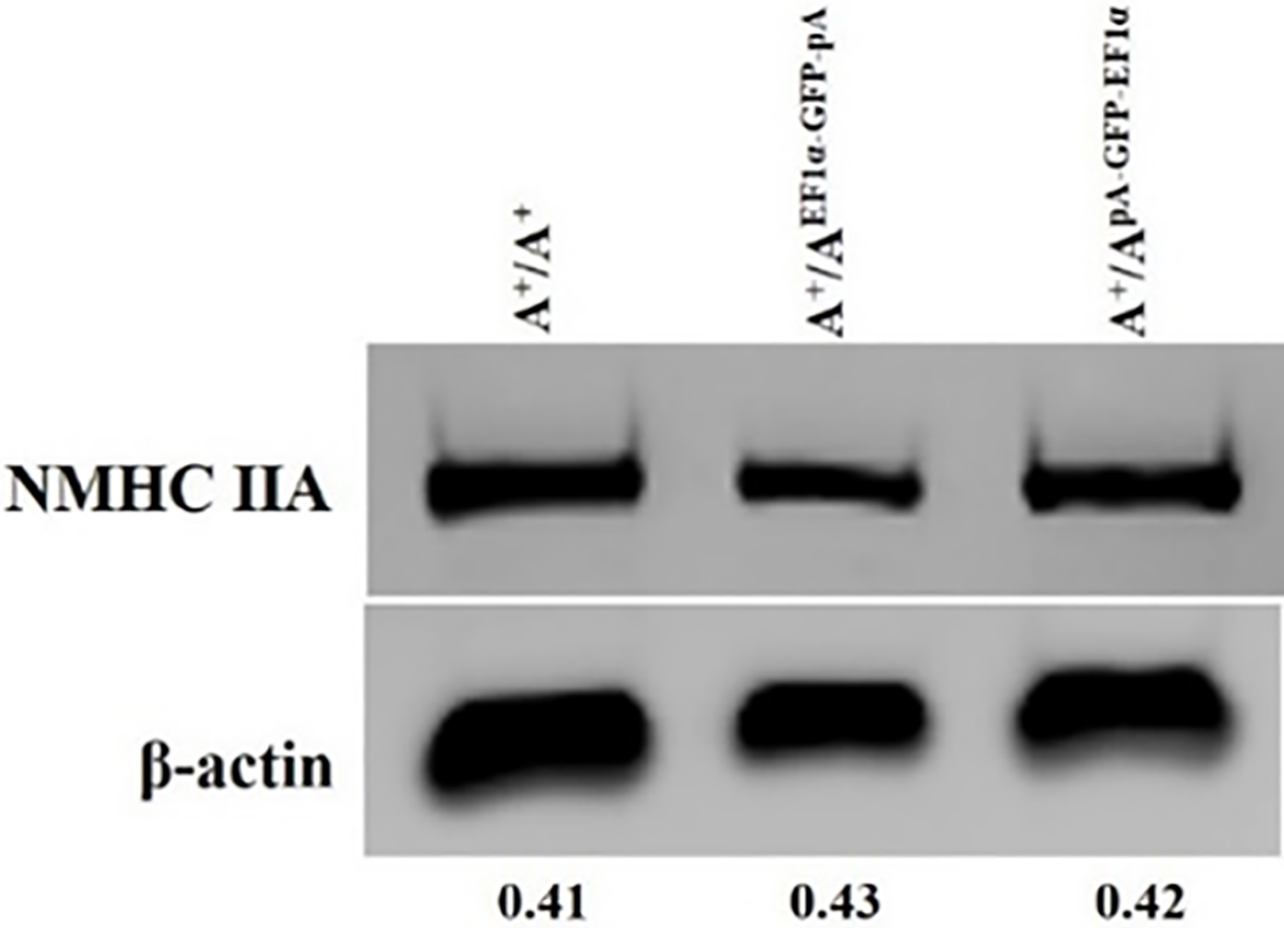
The expression level of the endogenous MYH9 gene is not affected by the insertion of exogenous gene(s) into intron 2. Immunoblot analysis of the expression levels of NMHC IIA in different cell lines probed with anti-NMHC IIA and anti- β-actin antibodies. Note the similar NMHC IIA levels in A^+^/A^EF1α-GFP-pA^ and A^+^/A^pA-GFP-EF1α^ cells to that of wild type cells. Representative results are from three repeats.

The expression of the endogenous MYH9 gene was examined, as all of its coding exons were preserved. Immunoblot assays demonstrated that the expression levels of the MYH9 gene in A^+^/A^KI EF1α-GFP-pA^, A^+^/A^KI pA-GFP-EF1α^ ES cells are completely comparable to that in A^+^/A^+^ ES cells, suggesting little or no effect of the inserted exogenous genes at the intron 2 site on the transcription of the MYH9 gene (Fig 5). Accordingly, its functions were preserved as well.

**Fig 6:**
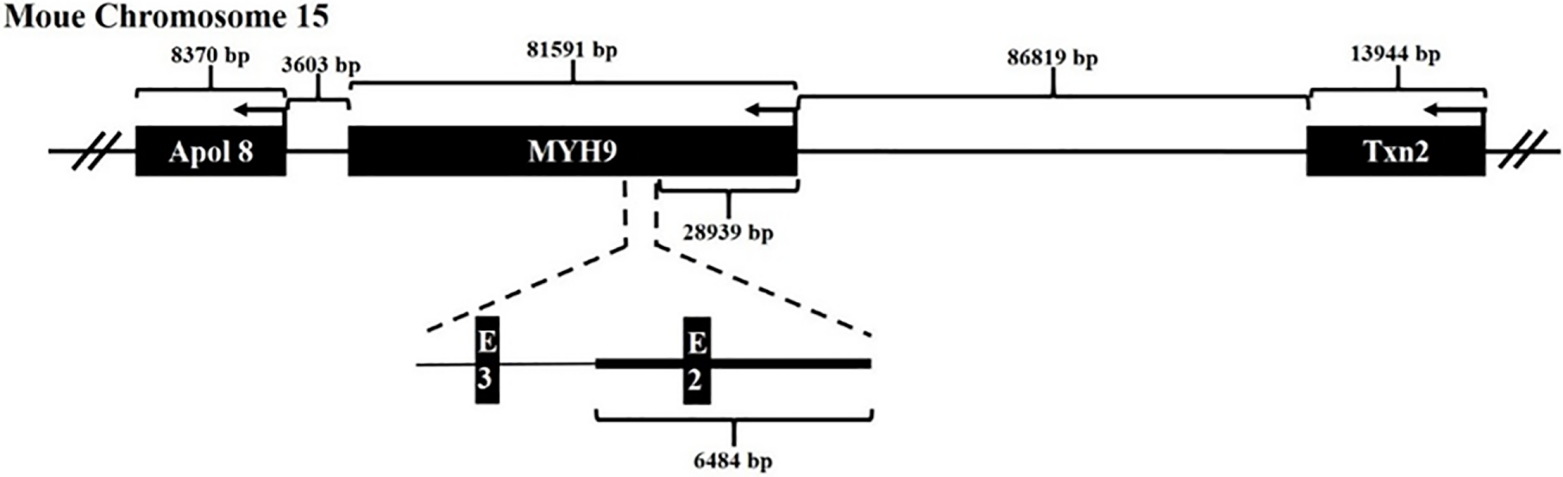
Gene context of MYH9 in mouse chromosome 15qE1. The MYH9 gene is located between Apol 8 and Txn2. The directions and distances between these genes are also indicated. The region used for creating homology arms in this study is shown below. The drawing is not to scale.

Finally, we analyzed the gene context of MYH9 in mouse chromosome 15. As indicated in Fig 6, the MYH9 gene is located at 15qE1 and flanked by the 5’ Txn2 gene which encodes the thioredoxin2 protein which is involved in redox control and protection against ROS-induced mitochondrial damage [32], and the 3’ Apol 8 gene encoding the Apolipoprotein L8 whose functions are less well studied. Additionally, it is apparent that the transcriptional regions of these genes are >50kb away from the targeting sites in exon 2 and intron2 of the MYH9 gene. The targeting sites are also >300 kb from any identified microRNA and cancer-related gene, and are located outside of ultra-conserved regions and long noncoding RNAs, Importantly, all heterozygous mice produced from the genetic replacement strategy shown in Fig 1 were normal, suggesting no influence on these flanking genes by targeted integration of exogenous cassettes at MYH9 exon 2. We therefore inferred that since the distance of the two targeting sites at exon 2 and intron 2 are in a range of <500 bp, these surrounding genes are also not affected by the targeted integration of exogenous cassettes at intron 2 of the MYH9 gene.

## Discussion

In the current study, we reported an ideal genome locus for targeted integration in ES cells and mice derived from these cells. Firstly, we found that gene targeting at the MYH9 exon 2 site has the highest HR efficiency (>95%) reported to date among similar approaches in use in mouse ES cells. Furthermore, this high efficiency was reproducible in the hands of different investigators [26]. In addition, mouse models produced from these positively targeted ES cell clones provide new insights into the functions of NM IIs in development; certain NMHC IIA mutations mimicked the phenotypes of MYH9-RD. Secondly, we presented evidence that exogenous gene(s) can be integrated into the MYH9 intron 2 site, while the high HR efficiency is maintained, and the sustainable and controllable expression of targeted inserted gene(s) is observed mainly dependent on the construct promoter(s). Finally, we confirmed that the expression and function of the MYH9 gene is well preserved when an exogenous gene(s) is integrated into the MYH9 intron 2 site. The results indicate that MYH9 may be another ideal safe harbor for targeted integration purposes.

A genetic replacement strategy is very useful for protein replacement therapy [33] and in studying the functions of genes which have different isoforms or clusters [34–40] and orthologs among distinct species as well [41–43]. In mammals, there are three different nonmuscle myosin II (NM II) isoforms (e.g. NM IIA, IIB, IIC), which have structural characteristics: each is composed of two pairs of light chains and a pair of heavy chains (e.g. NMHC IIA, IIB, IIC) encoded by the MYH9, MYH10, MYH14 genes, respectively. Each NM II contains two defined domains: an N-terminal globular region having MgATPase and actin-binding activities, and a C-terminal α-helical coiled-coil tail region responsible for filament assembly [44–45]. Complete ablation of NM IIA results in embryonic lethality at E6.5, while the ablation of NM IIB leads to embryonic lethality at E14.5, preventing further studies of the biological roles of these proteins in vivo [29,46]. To circumvent this problem, we utilized a genetic replacement strategy which involved the knockout of the endogenous MYH9 gene and the knock-in of exogenous expression cassette(s) encoding other NMHC IIs [25]. Indeed, this method is helpful in determining whether phenotypes are due to unique (isoform- or domain-specific) functions or tissue-dependent expression patterns with redundant functions of distinct NM IIs [25]. Our data therefore supports the effectiveness of the approach described by Carthon *et al*. 2005 [36]. It is worth noting that the prominent feature of increased gene targeting efficiency in mouse ES cells greatly facilitates the acquisition of desired ES clones and the generation of corresponding mouse models. This important advantage could be applied to investigate the isoform-specific functions of NM IIC in mouse early development and as well as the functions of other nonmuscle myosins.

The important functions of NM IIA are reflected in the embryonic lethality of knockout mice and demonstrated by its mutation-related diseases. Mutations in the MYH9 gene encoding NMHC IIA cause an autosomal-dominant disorder, namely MYH9-RD which has a complex phenotype characterized by macrothrombocytopenia with giant platelets and leukocyte inclusion bodies and syndromic forms combining these hematological features with deafness and/or nephropathy and/or cataracts [45,47–48]. At present, 49 MYH9 mutations have been reported to be associated with MYH9-RD [47]. However, the mechanism underlying MYH9-RD still remains to be determined. Two methods were used to generate MYH9-RD mouse models: one was to exploit cDNA encoding mutated R702C NMHC IIA to ablate and replace the endogenous gene, as described in Fig 1A and a previous study [26]; another was to mutate the R702C codon in the corresponding exon of the endogenous MYH9 gene [30]. Despite the difference in methodology, largely similar results were obtained. Given the high targeting efficiency at the MYH9 exon 2 and the utility of adding a fluorescent tag on cDNA encoding mutant NMHC IIA for real-time imaging, the former approach should be considered for generating MYH9-RD mouse models with other NMHC IIA mutations. These newly produced animal models will no doubt advance our understanding of the effects of NMHC IIA mutations on NM IIA functions and the underlying mechanisms of MYH9-RD.

Our data indicated that gene targeting using the vectors described in Fig 1A resulted in high-targeting efficiency, wherein the endogenous MYH9 gene was also disrupted. It suggests that genomic safe harbors are extragenic sites that are far away from a gene or genomic regulatory sequence, or an intragenic site (within a gene) whose ablation is tolerated [14]. Though the disruption of an MYH9 allele was tolerated by cells and mice [25,29], any impact or interference with the endogenous gene(s) should be avoided [3]. Given the effects of the chromatin state or chromosomal context on the integration of exogeneous genes into the locus in mouse ES cells and fertilized eggs [4,49], we therefore created a new set of targeting constructs that shifted the integration site into MYH9 intron 2 (Fig 2). Additionally, the exogenous cassette(s) was intended to be driven by its fused promoter(s) since the MYH9 gene promoter is not ubiquitous or tissue-specific due to its selective expression [45]. Interestingly, our efforts and results indicated: i) in spite of some reduction in efficiency compared to that of the MYH9 exon 2 site (over 90%), over 70% HR efficiency was observed at the MYH9 intron 2 site. The underlying reason for this might be due to a gradual increase to 800 μg/ml of G418 concentration for the former experiment vs immediate use of this concentration for the latter. ii)single copy exogeneous gene targeted integration into an allele of the MYH9 gene occurred similar to the observation of gene targeting at the MYH9 exon 2 site. It has been suggested that as far as the expression level is concerned, single copy integration is better than multicopy or random integration [49]. iii) whether the integration orientation of the exogenous expression cassette(s) was the same as or opposite to that of the MYH9 gene, the expression of these exogeneous genes relied only on the exogenous promoter without any obvious impact from surrounding endogenous gene(s) in particular, that of the endogenous MYH9 gene. This was true for both ubiquitous or tissue-specific promoters. Taken together, these results suggested that our targeted gene-of-interest into the MYH9 intron 2 site may have significant merit for transgenic mouse production.

One important criterion to classify a genomic site as a safe harbor site is minimal interference of the transgene with the rest of the genome [3]. Notably, the expression of the MYH9 gene did not show substantial variation after the integration of the exogenous cassette into its intron 2 site (Fig 5), suggesting that the MYH9 locus can tolerate the insertion of an exogenous gene. Additionally, it might be surprising that transcriptional interference from the endogenous MYH9 gene on transgene transcription or vice-versa is also absent, as opposed to previous reports [49,50–52]. Of note, the examination of the mouse MYH9 locus indicated that the transcription initiation sites of the MYH9 gene, its surrounding genes, as well as transgenes are far away from each other (>30kb) (Fig 6). This might explain the lack of transcription interference.

Since random integration of exogenous genes into the genome is associated with some limitations including insertional mutagenesis, dose-dependent effects or variegated transgene expression, numerous efforts have been made to find genomic sites/safe harbors which can facilitate overcoming these shortcomings. In this regard, Rosa26 is the first site to be identified as a a good genomic site for targeted integration of transgenes [20]. Up to now, over 130 Rosa26 locus-based knockin mouse lines have been generated [53]. Additionally, other safe harbors including AAVS1, HPRT, H11, in different species such as humans, mice, rats, rabbits, and pigs, have been identified and characterized [54, 55, 56, 57]. These safe harbors possess the general feature of supporting adequate transgenic expression without perturbing endogenous gene structure or function that make them suitable for versatile transgenic applications, including gain-of-function, loss-of-function, lineage-tracing, and gene therapy [53]. The MYH9 locus described in this study not only has the general properties of a safe harbor, but also displays unique characteristics. These include the pronounced HR efficiency and the capability of supporting both ubiquitous and tissue-specific gene expression depending on the properties of the fused promoter instead of the locus, similar to the Rosa26 locus [58]. Therefore, the MYH9 locus reported here increases the options for safe harbors for different transgenic purposes.

## Conclusion

We have discovered and characterized a new mouse genome locus suitable for gene site-specific integration. Using this MYH9 locus, we can explore the specific functions of different NM II isoforms or the mechanisms by which mutated NMHC IIA causes MYH9-RD, and insert exogenous genes for targeted integration purposes. Importantly, the MYH9 gene located >50 kilobases (kb) from the 5′ end of any gene, >300 kb from any identified microRNA and cancer-related gene, as well as being located outside ultra-conserved regions and long noncoding RNAs, together with high HR frequency and the expected expression patterns meets the requirements of a safe harbor. The MYH9 gene locus described here may therefore be considered both a novel and useful locus.

## Supporting information

S1 Table The primers used in this study. Note: The restriction sites are underlined and bolded.
(DOCX)

S1 Video Cardiomyocytes derived from A^+^/A^KI αMHC-Puro-IRES-GFP-pA^ ES cells displayed spontaneous beating following 14-days of directed differentiation. (AVI)
