# Supplementary material for: Identification and characterization of MYH9 locus for high efficient gene knock-in and stable expression in mouse embryonic stem cells
Source: PLoS One. 2018 Feb 13;13(2):e0192641. doi: 10.1371/journal.pone.0192641 (PMC5811019; doi:10.1371/journal.pone.0192641)
Supplement: S1 Table — Note: The restriction sites are underlined and bolded. (DOCX) [file pone.0192641.s001.docx]

**S1 Table.** The primers used in this study

| **Primer** | **Sequence (5’-3’ orientation)** | **Usage** |
| --- | --- | --- |
| **Primers related to gene targeting at MYH9 exon2 site** | | |
| MYH9Exon2L-F | ACGC**GTCGAC**GAAGTGAAGCTCCTGGCTTTG | Amplifying 5’arm |
| MYH9Exon2L-R | TCC**CCGCGG**GTGACTTGCGGCCAGGACCTAAG | Amplifying 5’arm |
| MYH9Exon2R-F | GG**GGTACC**GGCTCAGCAGGCTGCAGACAAGTACCTC | Amplifying 3’arm |
| MYH9Exon2R-R | CG**GGATCC**CAGCGGGGTAGGAAGCACGATG | Amplifying 3’arm |
| LP-F | GGATTGAACCTGAGGCTTTG | Amplifying left probe |
| LP-R | GAGGAGGAGTGCTTGCTGTG | Amplifying left probe |
| RP-F | TTTACCCACTGACTCATACCTC | Amplifying right probe |
| RP-R | TGTGTTCTCCCTCCAATTACTC | Amplifying right probe |
| P1 | TCATGTTCTGTCGTTGTTCC | Genotyping |
| P2 | CAGTGGGATAAAGAGACTCC | Genotyping |
| P3 | ACTTGTACAGCTCGTCCATGC | Genotyping |
| P4 | ATGAGGAAATTGCATCGCATTGTC | HR event identification |
| P5 | TGGGACTCCTGGGTTGAGGGTTTTGG | HR event identification |
| **Primers related to gene targeting at MYH9 intron2 site** | | |
| MAI2L-F | ACGC**GTCGAC**GAAGTGAAGCTCCTGGCTTTG | Amplifying 5’arm |
| MAI2L-R | TCC**CCGCGG**CTGCATGCAGGGAACAGAGGG | Amplifying 5’arm |
| MAI2R-F | AGCTTT**GTTTAAAC**GAAGATCAAGCTCCCACCTGC | Amplifying 3’arm |
| MAI2R-R | CG**GGATCC**GGAGGCTGAAGCCCTGCCCAG | Amplifying 3’arm |
| EF1a-F | CC**TTAATTAA**CGTGAGGCTCCGGTGCCCGTC | EF1α-GFP fragment |
| rGlobin pA-R | CC**TTAATTAA**CTGCAGGTCGAGGGATCTTCAT | EF1α-GFP fragment |
| PIG-F | ACGC**GTCGAC**ATGACCGAGTACAAGCCCACGGTG | Puro-IRES-GFP fragment |
| PIG-R | ACGC**GTCGAC**TTACTTGTACAGCTCGTCCATGCC | Puro-IRES-GFP fragment |
| P6 | ATGAGGAAATTGCATCGCATTGTC | HR event identification |
| P7 | GGAACCTCGATGCGCATACATAG | HR event identification |
| P8 | CCAAGGTGGAGGACATGGCAG | Genotyping |
| P9 | GCTACAGGGTCTGACCAGGTGGTAC | Genotyping |

Note: Underlined and bolded is the restriction site.
